# The Climatology of Neurasthenia

**Published:** 1901-06

**Authors:** 


### The Climatology of Neurasthenia.

Dr. F. S. Pearce, of Philadelphia, considers neurasthenia a nutritional disease of the central nervous system, and it is his opinion that, after paying due regard to both the predisposing and exciting causes, we must put the treatment upon a nutritional basis. To begin with, a diagnosis must be made between hysteria per se and neurasthenia or neurasthenia with hysterical complications.

In dealing with incipient cases of neurasthenia and in convalescent cases he believes climatology is of very great importance. The two extremes to be avoided are a low, windy, treeless country (such as Portland, Oregon), and one characterized by constantly quiet atmosphere and high altitude with low atmospheric pressure (such as most parts of New Mexico). An altitude of over 2000 feet is unsuitable for most cases. Sea air in a well wooded country, far enough removed from the coast to avoid fogs, furnishes an almost ideal condition for the neurasthenic invalid.—*Pennsylvania Medical Journal,* April, 1901.

